# Impact of Age and Sex Interaction on Post-Acute Sequelae of COVID-19: An Italian Cohort Study on Adults and Children

**DOI:** 10.3390/jcm12082924

**Published:** 2023-04-18

**Authors:** Matteo Puntoni, Susanna Esposito, Laura Patrizi, Chiara Maria Palo, Michela Deolmi, Giovanni Autore, Valentina Fainardi, Caterina Caminiti, on behalf of the University Hospital of Parma LONG-COVID Research Team

**Affiliations:** 1 Clinical & Epidemiological Research Unit, University Hospital of Parma, 43125 Parma, Italy; 2 Pediatric Clinic, Department of Medicine and Surgery, University of Parma, University Hospital of Parma, 43125 Parma, Italy; susannamariaroberta.esposito@unipr.it (S.E.);

**Keywords:** post-acute sequelae, COVID-19, sex hormones, age

## Abstract

Identifying factors predisposing individuals to post-acute sequelae of COVID-19 (PASC) would allow for the timely treatment of those vulnerable. Attention on the role of sex and age is growing, but published studies have shown mixed results. Our objective was to estimate the effect modification of age on sex as a risk factor for PASC. We analyzed data from two longitudinal prospective cohort studies on adult and pediatric subjects positive to SARS-CoV-2 infection that were enrolled between May 2021 and September 2022. Age classes (≤5, 6–11, 12–50, >50 years) were based on the potential role of sex hormones on inflammatory/immune and autoimmune processes. A total of 452 adults and 925 children were analyzed: 46% were female and 42% were adults. After a median follow-up of 7.8 months (IQR: 5.0 to 9.0), 62% of children and 85% of adults reported at least one symptom. Sex and age alone were not significantly associated to PASC, but their interaction was statistically significant (*p*-value = 0.024): the risk was higher for males aged 0–5 (females vs. males HR: 0.64, 95% CI: 0.45–0.91, *p* = 0.012) and for females aged 12–50 (HR: 1.39, 95% CI: 1.04–1.86, *p* = 0.025), especially those in the cardiovascular, neurological, gastrointestinal and sleep categories. Further research on PASC with regard to sex and age is warranted.

## 1. Introduction

Two years into the COVID-19 pandemic, considerable advances have been made in the understanding of acute COVID-19, its management and treatment, and effective vaccines have been developed in a historically short timeframe [1]. However, as the health crisis becomes less threatening, the impact of the disease is far from over. In fact, increasing numbers of people report prolonged symptoms after recovery from COVID-19, a condition often called long COVID, post-COVID-19 condition, or post-acute sequelae of COVID-19, (PASC) among other names [2,3]. Prevalence rates of PASC in adults and children reported in reviews and meta-analyses vary greatly, ranging from zero up to 70%, depending on the study design and methodological quality [4,5,6], SARS-CoV-2 variants and vaccination status [7,8,9], the definition used and considered symptoms [10,11], and the follow-up duration [12,13]. To date, the mechanisms causing PASC are still poorly understood, and the treatments and outcomes are still unknown [14]. Comprehensive research is therefore urgently needed with regard to this condition, including the identification of factors that can predispose an individual to its development, or instead have a protective effect [15,16]. This knowledge will enable the prompt identification of vulnerable subjects who can be closely monitored and promptly provided with the necessary care and support and given sufficient resources [17]. In this regard, growing attention is given to the potential role of sex and age as determinants of long COVID risk [18].

Although women exhibit a lower risk for severe acute infection and lower mortality rates than males, growing evidence suggests that they are at an increased risk of developing PASC compared to men [18,19,20]. A meta-analysis conducted by some of the authors of this paper [21], which included 20 studies and 13,340 adult patients (age range 40–70 years), found a statistically significant association of female sex with any symptoms (OR 1.52; 95% CI 1.27–1.82), with mental health symptoms (OR 1.67, 95% CI 1.21–2.29), and with fatigue (OR 1.54, 95% CI 1.32–1.79).

Age is also being considered as a determinant of long-term consequences of COVID-19. Concerning the adult population, some literature reviews have suggested that risk of developing long COVID increased with increasing age [2,22], but evidence on this point is contradictory. For instance, a large matched cohort study [23] including 486,149 adults with confirmed SARS-CoV-2 infection and 1,944,580 controls found that risk of reporting symptoms at ≥12 weeks after infection increased along a gradient of decreasing age. In the pediatric population, evidence seems to suggest that older age is associated with greater risk. In this regard, the retrospective cohort study by Kostev et al. [24], including 6568 children and adolescents, found that patients 13–17 years of age were more likely to be diagnosed with PASC compared with those aged ≤5 years (RR = 3.14). Similarly, in a prospective study by Osmanov et al. [25] on 518 patients ≤18 years old hospitalized with confirmed COVID-19, older age was associated with persistent symptoms: compared with children <2 years of age, those 6–11 years of age had an OR = 2.57 (95% CI 1.29–5.36) for persistent symptoms, and those 12–18 years of age had an OR = 2.52 (95% CI 1.34–5.01). 

Although sex and age have been investigated as separate determinants of long COVID, it is important to evaluate whether interactions between these two demographic parameters may influence risk. To the best of our knowledge, no such analysis has yet been performed. 

Therefore, the objective of our study was to explore whether PASC risk changes according to sex and age variations, in a population including adults and children, using standardized follow-up data collection protocols developed by the International Severe Acute Respiratory and Emerging Infection Consortium (ISARIC) Global Adult and Pediatric COVID-19 follow-up working groups [26]. In particular, we classified age groups in such a way as to verify the hypothesis of a sex hormone effect.

## 2. Materials and Methods

This study is reported based on the Strengthening the Reporting of Observational Studies in Epidemiology (STROBE) checklist for cohort studies [27].

### 2.1. Study Design, Population and Setting

This work analyzed data from two longitudinal prospective cohort studies on adult and pediatric subjects, conducted in the ISARIC framework, the methods of which were described in previous papers [28,29]. The analysis herein reported has the main objective of investigating whether an interaction between age and sex exists that modulates long COVID risk. 

Only individuals with positive polymerase chain reaction (PCR) confirmed SARS-CoV-2 infection were included in this study. Adults were patients hospitalized for COVID-19 who had been discharged to home approximately 3 months before and who had provided their informed consent. Children were subjects under 18 years of age with symptom onset within approximately 1–3 months and the consent of their parent, caregiver or guardian to participate in the study. The adult and pediatric subjects were identified from electronic medical records and the Local Health Information System at the host institutions.

Given the lack of a clinical definition of PASC at the time the ISARIC study protocols were designed, in this study we considered any post-acute symptoms reported by the patient at the time of follow-up that were not explained by underlying conditions and were not present before the COVID-19 infection [26].

The studies were coordinated by the University Hospital of Parma, a 1044 bed facility with a catchment area of >400,000 inhabitants located in northern Italy, an area severely hit by the first wave of the COVID-19 epidemic [30]. Subjects were enrolled from 3 May 2021 to 26 September 2022 at the University Hospital of Parma (adults and children) and other pediatric centers across Italy (Naples, Palermo, Catanzaro, Bari, Milan, Ferrara, Rome, Piacenza, Genoa). 

The studies were approved by the Area Vasta Emilia Nord (AVEN) Local Ethics Committee. The adult study took place on 13 April 2021 (protocol no. 196/2021/OSS/AOUPR), and the pediatric study took place on 30 November 2021 (protocol no. 952/2021/OSS/AOUPR). Participant/parental consent was sought during hospital discharge or by telephone interview during follow-up, and then confirmed at the first hospital accessed. 

### 2.2. Data Collection

For the initial and follow-up assessments, we adopted the Tier 1 ISARIC Long-term Follow-up Study Case Report Form (CRF) for adults, and version 1.3 of the COVID-19 Pediatric Case Control Follow-up form for pediatric subjects, both developed by the ISARIC Global COVID-19 follow-up working group and independently translated into Italian. 

The follow-up was planned to track PASC at 3-to-6-month intervals for up to 3 years.

Study data were collected and managed adopting REDCap electronic data capture tools (Vanderbilt University, Nashville, TN, USA) hosted at the University Hospital of Parma [31,32]. Italian translations of the abovementioned forms were used in the form of surveys which the enrolled subjects were asked to complete with the supervision of senior academic researchers and trained physicians.

The initial survey data collection was performed by a team of trained physicians or medical students by direct, face to face interview or by telephone interview. After the second interview, subjects were asked to complete the survey on their own through a web link sent by email. Participants unable to complete the survey online were assisted by telephone from the same trained medical students or physicians who had performed the first interview.

### 2.3. Outcome Measures

The main outcome measures were to describe the relationship and estimate the interaction between the two sexes (female vs. male) and four age classes (≤5, 6–11, 12–50, >50 years) in determining PASC during the follow-up period. The two lower age classes were identified based on data by Osmanov et al. [25], which indicate a greater risk for older individuals compared to those 0–5 years. The third cut-off was selected following recent evidence suggesting that women under the age of 50 exhibit a greater prevalence of PASC [33]. Symptoms were categorized into ten manifestations: musculoskeletal, cardiovascular, respiratory, neurological or cognitive dysfunction, dermatological, gastrointestinal, sensory, sleep, fatigue, and poor appetite or weight loss (Table S1). Symptom categorization was based on previously published literature and on discussions within the ISARIC working group [15,25].

### 2.4. Statistical Analysis

Descriptive statistics used included mean and standard deviation (SD), and median and interquartile range (IQR) for continuous variable frequency (percentage) for categorical variables. Reporting rates of symptoms among age classes and between males and females were compared using the Pearson chi square or Fisher’s exact tests. The non-parametric test by Cuzick et al. [34] was adopted to test for trends in reporting rates across age classes. 

The outcomes were measured in terms of event occurrence, considering an event to be the first symptom recorded in the observation period of any subject, and the association in terms of hazard ratio. The cumulative symptom-free survival was estimated with the Kaplan–Meier method, considering the time from the first survey to the first symptom recorded; censoring was applied to the last available follow-up time point (last survey compiled) in the absence of symptoms. A log-rank test was used to test for differences among age classes or between males and females. Hazard ratios were calculated using a Cox proportional hazards regression model, and proportionality was checked using Schoenfeld residuals. Median follow-up time was calculated by adopting the reverse Kaplan–Meier method [35]. Forest plots were used to summarize the hazard ratios of each post-COVID-19 manifestation on females compared to males stratified by age classes. We performed tests for the interaction between sex and age, adding the specific interaction term within the Cox models. 

A subpopulation treatment effect pattern plot methodology (STEPP) [36] was used to explore and display the cumulative reporting rate of post-COVID symptoms in females compared to males and along the continuous scale of age by using overlapping subject subgroups. 

To determine the robustness of our assessments, we decided to perform two post-hoc sensitivity analyses. Firstly, we reanalyzed data of subjects aged ≥18 year (adults hospitalized) and <18 (pediatric outpatients) separately. Secondly, we reanalyzed the data excluding the first survey on pediatric subjects, which was completed closer to symptom onset for children than for adults.

Due to the exploratory nature of subgroup analyses, we did not apply any adjustment for multiplicity. We included all participants for whom the variables of interest were available in the final analysis, without imputing missing data. The results are shown with 95% confidence intervals (95% CI), and statistical significance was considered for two-sided *p* values < 5%. All analyses were performed using STATA version 17.0 (StataCorp, College Station, TX, USA).

## 3. Results

**Figure 1:**
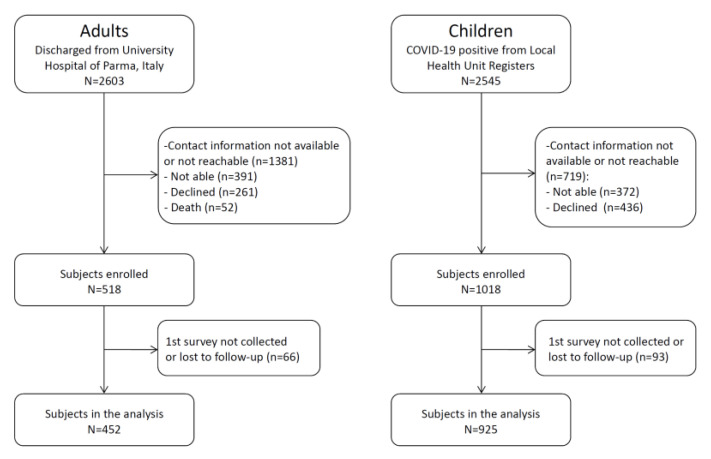
Flow-chart diagram of the cohorts.

A total of 2603 adults and 2545 children were eligible for the study; 1222 (47%) and 1826 (72%) could be contacted, and 518/1222 (42%) and 1018/1826 (56%) were enrolled. Overall, 452/518 (87%) adults and 925/1018 (91%) children completed at least the initial survey and were included in the analyses (Figure 1).

The timings of the initial and the follow-up surveys are shown in Table S2; in adults the first survey was taken at a median of 3.3 months from hospital discharge, and children (or parents) were initially interviewed after a median of 2.4 months from the first COVID-19 symptom. The main demographics of the enrolled subjects are shown in Table 1: 46% were female, 43% were adults, and 48% were children; the median age in adults was 59 years (IQR, 50–68), while it was 8 years (IQR, 6–11) in children.

During the observation period, 62% (575/925) of children and 85% (383/452) of adults reported at least one symptom (Fisher’s exact test *p* < 0.001). During the first survey, 263/925 (28%) children and 319/452 (71%) adults reported more than one symptom. Furthermore, in the second survey, 34% (157/458) of children and 64% (226/351) of adults reported persistent symptoms.

The categorized reported symptoms are summarized in Table S3 by sex and age group. The most common symptoms reported in children were respiratory (36%), neurological (27%), fatigue (20%), and gastrointestinal (19%); in adults, neurological (66%), fatigue (64%), musculoskeletal pain (63%) and respiratory (57%) symptoms were the most common. 

Another variable collected in the survey was anti-COVID-19 vaccination status. This data is shown in Table S4: 360 (80%) of the hospitalized adults and 198 (21%) of the pediatric outpatients were vaccinated with at least one dose.

Overall, the subjects were followed for a median of 7.8 months (IQR: 5.0 to 9.0). Time to first reported post COVID-19 symptom analysis showed that females (any age) did not have a significantly higher risk than males (Log-rank *p* = 0.289, Figure S1). Similarly, no difference among age classes was observed (Log-rank *p* = 0.273, Figure S2).

**Figure 2:**
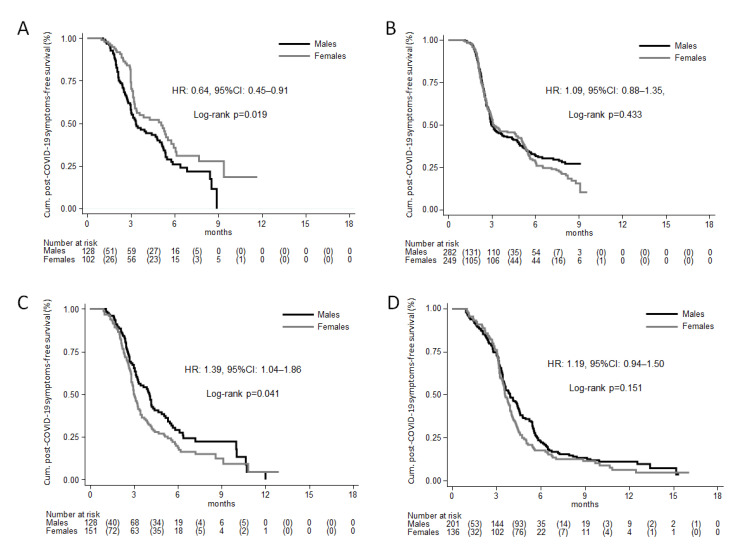
Kaplan-Meier estimates of cumulative reported frequency of post-COVID-19 symptoms, by sex, (**A**) in subjects 0–5 years of age, (**B**) 6–11 years of age, (**C**) 12–50 years of age, and (**D**) >50 years of age. The numbers in parentheses represent the number of symptoms that were reported within each time interval, by sex. The estimate of the hazard ratio (HR) was based on a Cox proportional hazards regression model, adjusted for age.

The subgroup analysis, which was conducted to investigate the interaction between sex and age, showed significantly different risk estimates in females vs. males by age class (*p*-value for sex × age interaction = 0.024). Specifically, the difference was statistically significant in the age class of 0–5 years of age, where the risk was higher for males (HR: 0.64, 95% CI: 0.45–0.91, *p* = 0.012, Figure 2A) and in subjects 12–50 years of age, while the risk was higher for females (HR: 1.39, 95% CI: 1.04–1.86, *p* = 0.025, Figure 2C); no difference was observed in subjects aged 6–11 year (HR: 1.09, 95% CI: 0.88–1.35, *p* = 0.436, Figure 2B) and in subjects >50 years of age (HR: 1.19, 95% CI: 0.94–1.50, *p* = 0.155, Figure 2D). 

**Figure 3:**
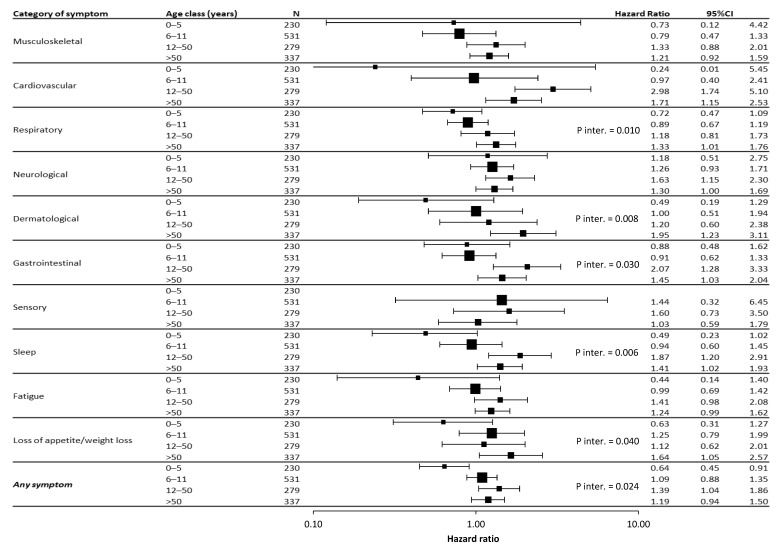
Forest plot of the risk estimate (hazard ratio, HR) of post-COVID-19 symptoms for females vs. males in each symptom category, stratified by age group. HRs are adjusted for age. Abbreviations: *p*-inter., *p*-value for the sex × age interaction term in the Cox model. Notes: HR for sensory symptoms in the 0–5 year group was not estimable because of a lack of events; data are from all of the surveys collected.

The forest plot in Figure 3 graphically summarizes the hazard ratios of females compared to males in each considered age class for each symptom category and overall for “Any Symptom”.

The interaction between sex and age was statistically significant overall for Any Symptom (*p* = 0.024), and in particular for respiratory (*p* = 0.010), dermatological (*p* = 0.008), and gastrointestinal (*p* = 0.030) symptoms, sleep problems (*p* = 0.006), and the loss of appetite or weight loss (*p* = 0.040). Similarly, the analysis of hazard ratios for each symptom category and age range (Figure 3) suggested that the risk was higher for males in the 0–5 year age class for almost all categories, but this was not statistically significant; and for females in the 12–50 year class for almost all categories, and the association was statistically significant for cardiovascular, neurological, and gastrointestinal symptoms, and sleep. Lower but statistically significant HRs were also found for women >50 years of age in all categories except for musculoskeletal, sensory, and fatigue.

**Figure 4:**
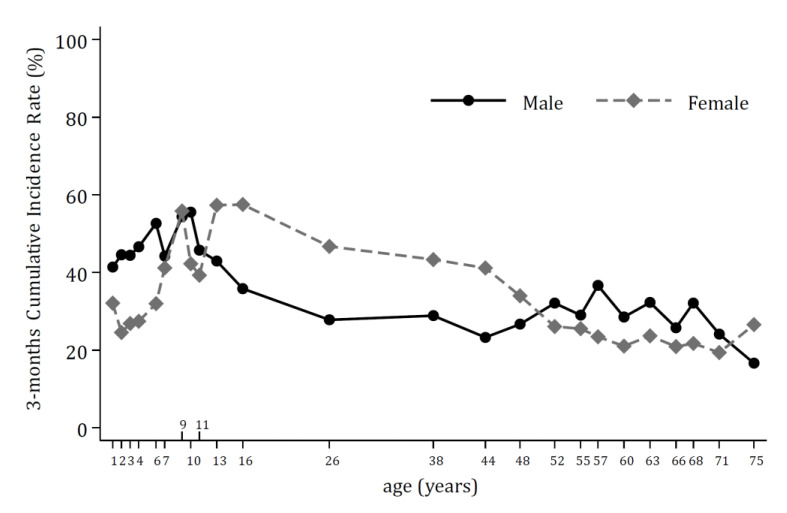
Subpopulation Treatment Effect Pattern Plot (STEPP) of the 3-month cumulative incidence rate of the reported symptoms in males (solid black line) and females (dotted grey line). The plot was drawn adopting the sliding window pattern, including *n* = 100 subjects in each subpopulation and *n* = 75 subjects in common among consecutive subpopulations, implementing 2500 permutations of the covariate age.

Finally, adopting the STEPP graphical technique (Figure 4), differences between the two sexes by age class appear to confirm our findings: the 3-months cumulative incidence rate of any post-COVID-19 symptoms is higher for females vs. men in the of 12–50 years of age range, while it seems lower below 6/7 years of age (interaction *p*-value based on cumulative incidence estimates = 0.012).

### Sensitivity Analyses

The analysis considering hospitalized adults and pediatric outpatients separately (Figures S3–S5) confirmed the higher risk estimates for females in the 12–17 and 18–50 age ranges as compared to males and to other age classes. With regard to the further sensitivity analysis, it was carried out by excluding the data obtained from the first survey in pediatric patients (Figures S6 and S7), and despite the expected wider confidence intervals, the direction of the estimates in the age groups considered was almost superimposable.

## 4. Discussion

This paper reported the analysis of two follow-up cohorts of 452 adults and 925 children with a laboratory confirmed diagnosis of SARS-CoV-2 infection. This work was conducted in the framework of an international initiative led by ISARIC, and used the ISARIC Global COVID-19 follow-up protocol for adults and children. Numerous publications have been produced [6,18] mainly aimed at determining the frequencies of the reporting of PASC and characterizing the factors associated with their occurrence. Some of these studies have investigated the influence of sex or age separately, and we only know of one study [37] which described a higher prevalence of PASC in women <50 years of age among adult patients discharged from the hospital. Therefore, to better understand the factors influencing the magnitude or direction of the relationship between virus exposure and the onset of PASC, our study aimed to examine two potential moderators, sex and age, identified on the basis of previous research which, however, highlighted conflicting results [6,18].

To the best of our knowledge, this is the first study focused on assessing how the risk of PASC is modulated by the interaction between sex and age in children and adults. While our overall analysis did not detect any effect considering these two factors separately, the analysis of the interaction suggests that women aged between 12 and 50 years of age exhibited a 40% higher risk than men for PASC; in particular, the risk was three times higher for cardiovascular symptoms, and twice as high for gastrointestinal and sleep problems. Sex differences in PASC have been attributed to biological (i.e., hormones and immune responses), and sociocultural (i.e., sanitary-related behaviors, psychological stress, and inactivity) aspects [18,38]. In particular, the higher prevalence of PASC in women between 12–17 and 18–50 years is an important and supporting clue of the role of sex hormones, also considering that the mean age of natural menopause is 51 years [33]. This hypothesis is plausible; however, considering that hormone levels vary considerably within such a wide age range, it does require further investigation, which was not feasible in our study due to the lack of adequate sample size in this age range.

Another finding emerging from our analysis is that among pediatric subjects, the risk for PASC appeared to be higher for males compared to females in the 0–5-year age range, although no significant associations were found in the analysis of individual symptom categories, which was also due to the high variability of risk estimates. We could not find studies exploring these aspects in the literature, and it is difficult to give a plausible explanation for this finding. It should also be taken into account that for children in this age class, responses were provided by parents, therefore the results should be interpreted with caution.

This study has some strengths, which include the use of standardized ISARIC Long-term Follow-up Study CRFs, the enrolment of both adults and children combined in the analysis of data, and a relatively large sample size of people attending multiple clinical centers.

Some limitations of the study must also be considered. Firstly, we did not include a control arm, and prevalence data may have been overestimated, as shown in the meta-analysis by Behnood et al. [6]; similarly, the combined effect between sex and age observed in this work may be due to factors other than COVID-19. Secondly, as our results were obtained with subgroup analyses without adjustments for multiple comparisons, which exhibit known limitations [39], they should be considered with caution and verified in ad hoc studies. Thirdly, this study is based on self-reported symptoms, and data may be biased due to psychological and sociocultural factors. Fourthly, the risk of potential bias should be pointed out. Selection bias may be present: for instance, individuals with symptoms may be more likely to respond to the survey. Notably, among potentially eligible subjects, only 42% of adults and 56% of children participated. The remaining individuals could not be contacted, did not give their informed consent, or were judged to be unable to take part in the survey. Furthermore, the proportion of responders decreased in subsequent follow-up surveys, which, however, was expected due to the voluntary nature of survey completion and web-based self-administration [40]. Again, it is possible that dropouts may be more frequent among individuals without important symptoms, or in those whose symptoms improved.

## 5. Conclusions

This study adds to the evidence on the importance of sex as a risk factor for PASC, but only in specific age ranges. In particular, the elevated risk found in women 12–50 years old emphasizes the need to further investigate the role of sex hormones on inflammatory/immune and autoimmune processes in greater depth. Future research on PASC should be considered from the perspective of sex and age. Taking these differences into account in the diagnosis, the prevention and treatment of COVID are critical steps towards precision medicine.

## Figures and Tables

**Table 1:** Demographics.

	Adults (*n* = 452)	Children (*n* = 925)	All (*n* = 1377)
Sex, *n* (%)			
Male	257 (57)	482 (52)	739 (54)
Female	195 (43)	443 (48)	638 (46)
Age, years			
mean (SD)	58.4 (13.7)	8.0 (4.2)	24.6 (25.2)
median (IQR)	59 (50–68)	8 (6–11)	11 (7–50)
min–max	19–86	0–17	0–86
Age classes, years			
0–5	-	230 (25)	230 (17)
6–11	-	531 (57)	531 (39)
12–17	-	164 (18)	164 (12)
18–50	115 (25)	-	115 (8)
51–64	172 (38)	-	172 (13)
65+	165 (37)	-	165 (12)

Abbreviations: SD, standard deviation; IQR, interquartile range.

## Data Availability

Data supporting the findings of this study are available from the corresponding author, M.P., upon reasonable request.

## Supplementary Materials

The following supporting information can be downloaded at: https://www.mdpi.com/article/10.3390/jcm12082924/s1: Table S1. Categorization of post-COVID-19 symptoms; Table S2. Number of subjects interviewed and the timing of the interviews in adults and children; Table S3. Frequency of post-COVID-19 symptoms in categories, by sex and age classes; Table S4: Vaccination status in adults and children analysed, at the time of the last survey; Figure S1: Kaplan-Meier estimates of cumulative reported frequency of post-COVID-19 symptoms in the whole cohort, by sex. Figure S2: Kaplan-Meier estimates of cumulative reported frequency of post-COVID-19 symptoms in the whole cohort, by age class; Figure S3: Kaplan-Meier estimates of cumulative reported frequency of post-COVID-19 symptoms, by sex; Figure S4: Forest plot of the risk estimate (hazard ratio, HR) of post-COVID-19 symptoms for females vs. males in each symptom category, stratified by age group, in pediatric outpatients.; Figure S5: Forest plot of the risk estimate (hazard ratio, HR) of post-COVID-19 symptoms for females vs. males in each symptom category, stratified by age group, in hospitalized adults; Figure S6: Forest plot of the risk estimate (hazard ratio, HR) of post-COVID-19 symptoms for females vs. males in each symptom category, stratified by age group, excluding data from the 1st survey of pediatric outpatients; Figure S7: Forest plot of the risk estimate (hazard ratio, HR) of post-COVID-19 symptoms for females vs. males in each symptom category, stratified by age group, in pediatric outpatients, excluding data from the 1st survey.
