# Supplementary material for: Impact of Age and Sex Interaction on Post-Acute Sequelae of COVID-19: An Italian Cohort Study on Adults and Children
Source: J Clin Med. 2023 Apr 18;12(8):2924. doi: 10.3390/jcm12082924 (PMC10144783; doi:10.3390/jcm12082924)

Supplemental material:

Table S1. Categorisation of post-COVID-19 symptoms

Table S2. Number of subjects interviewed and timing of the interviews in adults and children

Table S3. Frequency of post-COVID-19 symptoms in categories, by sex and age classes*, males, n (%) / females, n (%)*

Table S4. Vaccination status in adults and children analysed, at the time of the last survey, n (%)

Figure S1: Kaplan-Meier estimates of cumulative reported frequency of post-COVID-19 symptoms in the whole cohort, by sex. Numbers in parentheses represent the number of symptoms that were reported within each time interval, by sex.

Figure S2: Kaplan-Meier estimates of cumulative reported frequency of post-COVID-19 symptoms in the whole cohort, by age class. Numbers in parentheses represent the number of symptoms that were reported within each time interval, by age class.

Figure S3: Kaplan-Meier estimates of cumulative reported frequency of post-COVID-19 symptoms, by sex, (A) in subjects aged 0-5y, (B) 6-11y, (C) 12-17y, (D) 18-50y, (E) 51-64y and (F) 65+y. Numbers in parentheses represent the number of symptoms that were reported within each time interval, by sex. The estimate of the hazard ratio (HR) was based on a Cox proportional hazards regression model, adjusted for age.

Figure S4: Forest plot of the risk estimate (hazard ratio, HR) of post-COVID-19 symptoms for females vs. males in each symptom category, stratified by age group, in pediatric outpatients. HRs are adjusted for age. Abbreviations: P-inter., p-value for the sex*age interaction term in the Cox model. Note: HR for sensory symptoms in 0-5y group was not estimable because of lack of events; data are from all the surveys collected.

Figure S5: Forest plot of the risk estimate (hazard ratio, HR) of post-COVID-19 symptoms for females vs. males in each symptom category, stratified by age group, in hospitalized adults. HRs are adjusted for age. Abbreviations: P-inter., p-value for the sex*age interaction term in the Cox model; data are from all the surveys collected.

Figure S6: Forest plot of the risk estimate (hazard ratio, HR) of post-COVID-19 symptoms for females vs. males in each symptom category, stratified by age group, excluding data from the 1st survey of pediatric outpatients. HRs are adjusted for age. Abbreviations: P-inter., p-value for the sex*age interaction term in the Cox model. Note: HR for sensory symptoms in 0-5y group was not estimable because of lack of events.

Figure S7: Forest plot of the risk estimate (hazard ratio, HR) of post-COVID-19 symptoms for females vs. males in each symptom category, stratified by age group, in pediatric outpatients, excluding data from the 1st survey. HRs are adjusted for age. Abbreviations: P-inter., p-value for the sex*age interaction term in the Cox model. Note: HR for sensory symptoms in 0-5y group was not estimable because of lack of events.

Table S1. Categorisation of post-COVID-19 symptoms.

**Category**	**Symptoms**
Musculoskeletal	Joint pain or swelling, Persistent muscle pain, Weakness arms/legs or muscle weakness.
Cardiovascular	Palpitations (heart racing), Variations in heart rate (tachycardia or bradycardia).
Respiratory	Shortness of breath/breathlessness, Pain on breathing, Persistent cough, Chest pain, Nasal congestion/rhinorrea.
Neurological and cognitive dysfunction	Cannot fully move or control movement, Problems with balance, Tremor/shakiness, Seizures, Tingling feeling/“pins and needles”, Confusion/lack of concentration, Problems speaking or communicating, Fainting/ blackouts, Dizziness/ light headedness, Problems seeing/blurred vision, Headache, problems swallowing or chewing, ringing in ears, can’t feel one side of body/face.
Dermatological	Hair loss, Skin rash, Lumpy lesions on toes,
Gastrointestinal	Constipation, Diarrhoea, Stomach/ abdominal pain, Vomiting
Sensory	Loss of smell, Loss of taste
Sleep	Problems sleeping, Hypersomnia (children only)
Fatigue	Fatigue
Poor appetite/Weight loss	Poor appetite, Weight loss (adults only)

Table S2. Number of subjects interviewed and timing of the interviews in adults and children

	No. subjects surveyed	time from discharge, *months, median (IQR)*
Adults (n=518):		
1st survey (expected 1-3 mos after hospital discharge)	450	3.3 (2.4-4.3)
2nd survey (expected 3 mos after 1st survey)	362	6.9 (5.9-8.4)
3rd survey (expected 6 mos after 1st survey)	227	9.9 (8.7-11.4)
4th survey (expected 9 mos after 1st survey)	125	12.4 (11.6-13.2)
5th survey (expected 12 mos after 1st survey)	81	15.3 (14.6-16.3)
Children (n=1,010):		time from 1st symptom, *months, median (IQR)*
1st survey (expected 1-3 mos after 1st COVID symptom)	905	2.4 (2.0-2.9)
2nd survey (expected 3-6 mos after 1st COVID symptom)	559	5.4 (5.0-5.9)
3rd survey (expected 6-12 mos after 1st COVID symptom)	328	8.4 (8.0-8.8)

Abbreviations: IQR, interquartile range

Table S3. Frequency of post-COVID-19 symptoms in categories, by sex and age classes, *males, n (%) / females, n (%)**

	0-5y (n=128/102)	6-11y (n=282/249)	12-50y (n=128/151)	>50y (n=201/136)	All children (n=482/443)	All adults (n=257/195)
Musculoskeletal pain	3 (2.3) / 2 (2.0) p>0.99	33 (11.7) / 25 (10.0) p=0.579	41 (32.0) / 55 (36.4) p=0.451	121 (60.2) / 89 (65.4) p=0.360	44 (9.1) / 40 (9.0) p>0.99	154 (59.9) / 131 (67.2) p=0.117
Cardiovascular	2 (1.6) / 1 (1.0) p>0.99	10 (3.6) / 9 (3.6) p>0.99	20 (15.6) / 49 (32.5) p=0.001	49 (24.4) / 50 (36.8) p=0.015	14 (2.9) / 26 (5.9) p=0.034	67 (26.1) / 83 (42.6) p<0.001
Respiratory	57 (44.5) / 39 (38.2) p=0.349	102 (36.2) / 84 (33.7) p=0.585	47 (36.7) / 65 (43.1) p=0.327	110 (54.7) / 90 (66.2) p=0.042	174 (36.1) / 155 (35.0) p=0.732	142 (55.3) / 123 (63.1) p=0.102
Neurological and cognitive dysfunction	11 (8.6) / 11 (10.8) p=0.654	77 (27.3) / 89 (35.7) p=0.039	53 (41.4) / 88 (58.3) p=0.006	125 (62.2) / 98 (72.1) p=0.062	107 (22.2) / 145 (32.7) p<0.001	159 (61.9) / 141 (72.3) p=0.021
Dermatological	14 (10.9) / 6 (5.9) p=0.240	18 (6.4) / 17 (6.8) p=0.862	16 (12.5) / 19 (12.6) p>0.99	33 (16.4) / 39 (28.7) p=0.010	35 (7.3) / 28 (6.3) p=0.603	46 (17.9) / 53 (27.2) p=0.022
Gastrointestinal	26 (20.3) / 18 (17.7) p=0.736	57 (20.2) / 50 (20.1) p>0.99	26 (20.3) / 53 (35.1) p=0.008	68 (33.8) / 62 (45.6) p=0.031	92 (19.1) / 86 (19.4) p=0.934	85 (33.1) / 97 (49.7) p<0.001
Sensory	2 (1.6) / 0 (-) p=0.504	3 (1.1) / 4 (1.6) p=0.711	10 (7.8) / 18 (11.9) p=0.319	30 (14.9) / 21 (15.4) p>0.99	9 (1.9) / 10 (2.3) p=817	36 (14.0) / 33 (16.9) p=0.429
Sleep	25 (19.5) / 10 (9.8) p=0.044	43 (15.3) / 38 (15.3) p>0.99	30 (23.4) / 60 (39.7) p=0.005	82 (40.8) / 72 (52.9) p=0.034	81 (16.8) / 80 (18.1) p=0.664	99 (38.5) / 100 (51.3) p=0.007
Fatigue	10 (7.8) / 4 (3.9) p=0.274	62 (22.0) / 57 (22.9) p=0.835	51 (39.8) / 76 (50.3) p=0.092	120 (59.7) / 96 (70.6) p=0.049	88 (18.3) / 98 (22.1) p=0.163	155 (60.3) / 135 (69.2) p=0.060
Poor appetite/Weight loss	23 (18.0) / 12 (11.8) p=0.203	34 (12.1) / 39 (15.7) p=0.256	19 (14.8) / 27 (17.9) p=0.521	37 (18.4) / 40 (29.4) p=0.024	65 (13.5) / 68 (15.4) p=0.453	48 (18.7) / 50 (25.6) p=0.084
Any symptom (at least 1)	*83 (64.8) / 53 (52.0) p=0.059*	*173 (61.4) / 166 (66.7) p=0.207*	*83 (64.8) / 114 (75.5) p=0.065*	*166 (82.6) / 120 (88.2) p=0.167*	*293 (60.8) / 282 (63.7) p=0.378*	*212 (82.5) / 171 (87.7) p=0.147*

Abbreviations: y, years old. *Fisher exact p-values are shown.

Table S4. COVID-19 vaccination status in adults and children analysed, at the time of the last survey, n (%)

	Hospitalized adults (n=452)	Pediatric outpatients (n=925)
Yes (at least 1 dose)	360 (79.7)	198 (21.4)
2 doses	244 (54.0)	106 (11.5)
3 doses (“booster”)	130 (28.8)	26 (2.8)
No	88 (19.5)	725 (78.4)
“Don’t know” or not compiled	4 (0.9)	2 (0.2)

Supplementary figures:

Figure S1: Kaplan-Meier estimates of cumulative reported frequency of post-COVID-19 symptoms in the whole cohort, by sex. Numbers in parentheses represent the number of symptoms that were reported within each time interval, by sex.

Figure S2: Kaplan-Meier estimates of cumulative reported frequency of post-COVID-19 symptoms in the whole cohort, by age class. Numbers in parentheses represent the number of symptoms that were reported within each time interval, by age class.

Figure S3: Kaplan-Meier estimates of cumulative reported frequency of post-COVID-19 symptoms, by sex, (A) in subjects aged 0-5y, (B) 6-11y, (C) 12-17y, (D) 18-50y, (E) 51-64y and (F) 65+y. Numbers in parentheses represent the number of symptoms that were reported within each time interval, by sex. The estimate of the hazard ratio (HR) was based on a Cox proportional hazards regression model, adjusted for age.

Figure S4: Forest plot of the risk estimate (hazard ratio, HR) of post-COVID-19 symptoms for females vs. males in each symptom category, stratified by age group, in pediatric outpatients. HRs are adjusted for age. Abbreviations: P-inter., p-value for the sex*age interaction term in the Cox model. Note: HR for sensory symptoms in 0-5y group was not estimable because of lack of events.

Figure S5: Forest plot of the risk estimate (hazard ratio, HR) of post-COVID-19 symptoms for females vs. males in each symptom category, stratified by age group, in hospitalized adults patients. HRs are adjusted for age. Abbreviations: P-inter., p-value for the sex*age interaction term in the Cox model.

Figure S6: Forest plot of the risk estimate (hazard ratio, HR) of post-COVID-19 symptoms for females vs. males in each symptom category, stratified by age group, excluding data from the 1st survey of pediatric outpatients. HRs are adjusted for age. Abbreviations: P-inter., p-value for the sex*age interaction term in the Cox model. Note: HR for sensory symptoms in 0-5y group was not estimable because of lack of events.

Figure S7: Forest plot of the risk estimate (hazard ratio, HR) of post-COVID-19 symptoms for females vs. males in each symptom category, stratified by age group, in pediatric

outpatients, excluding data from the 1st survey. HRs are adjusted for age. Abbreviations: P-inter., p-value for the sex*age interaction term in the Cox model. Note: HR for sensory symptoms in 0-5y group was not estimable because of lack of events.

Figure S1

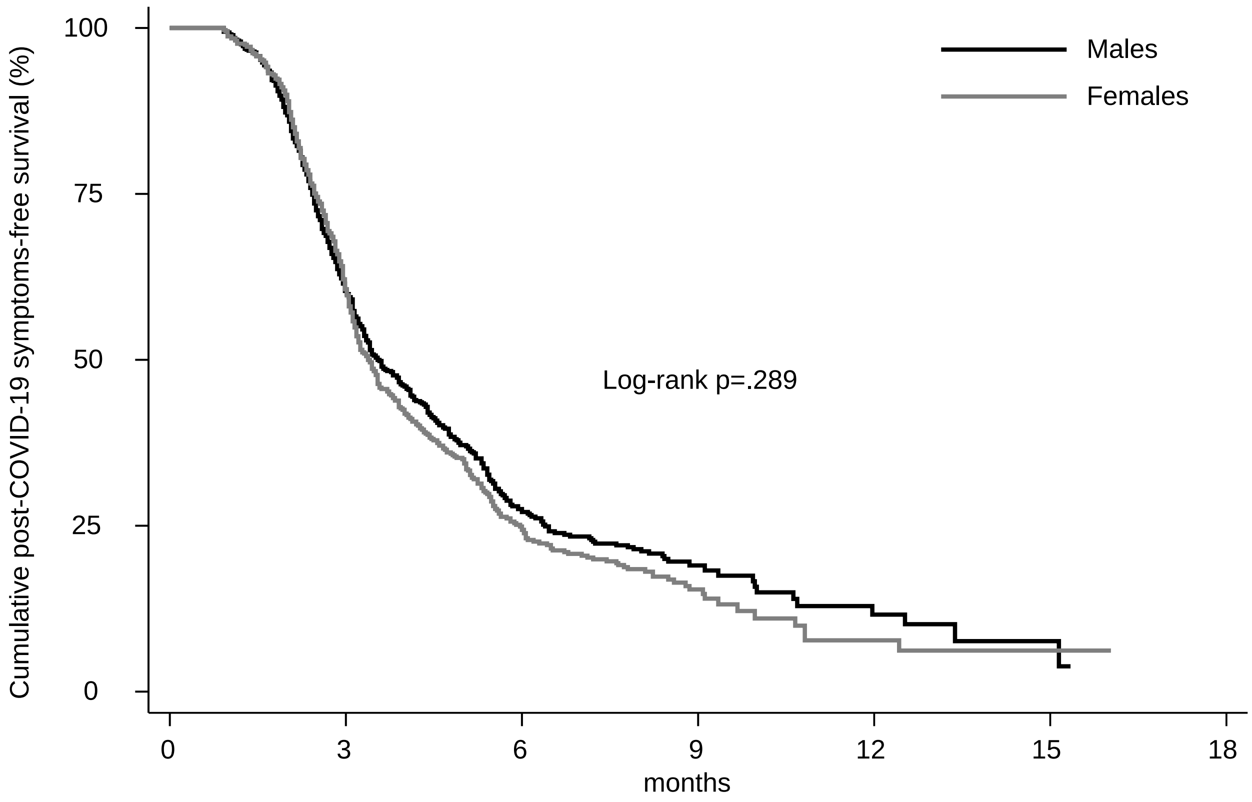

Number at risk

Males	739	(275)	381	(189)	124	(30)	28	(8)	9	(2)	2	(1)	0
Females	638	(235)	327	(178)	99	(31)	26	(8)	5	(1)	1	(0)	0

Figure S2

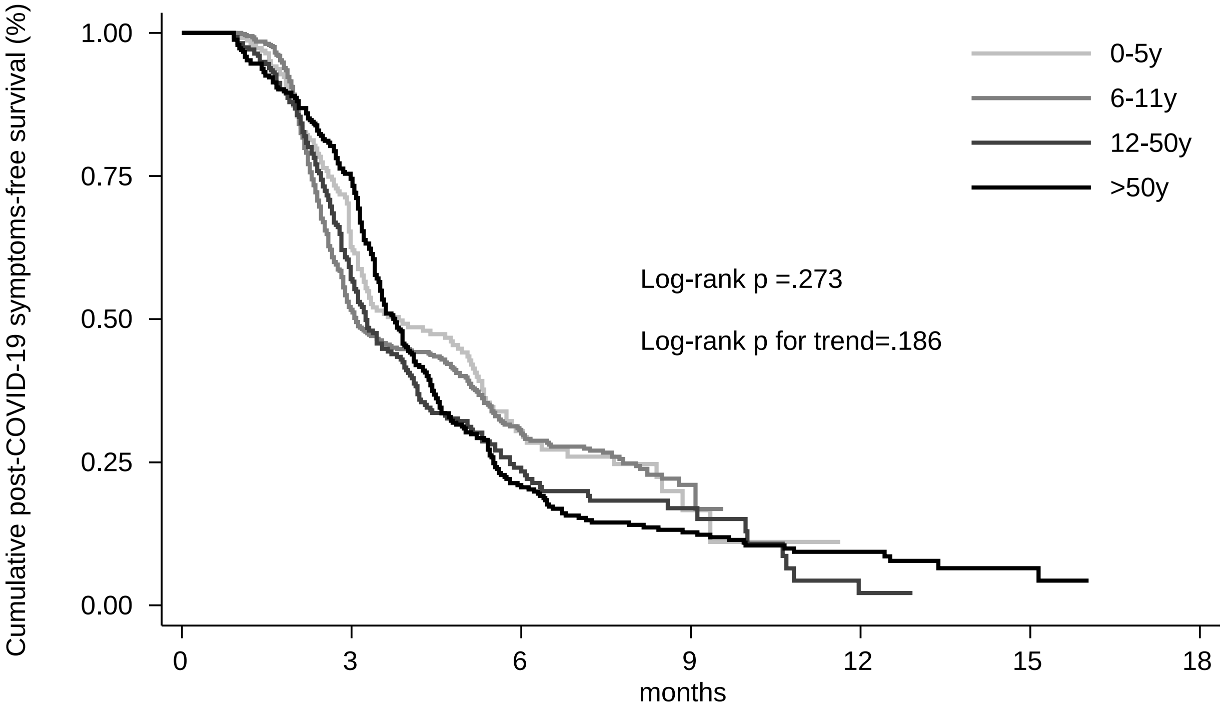

Number at risk

0-5y	230	(77)	115	(50)	31	(8)	5	(1)	0	(0)	0	(0)	0
6-11y	531	(236)	216	(79)	98	(23)	9	(1)	0	(0)	0	(0)	0
12-50y	279	(112)	131	(69)	37	(9)	10	(7)	1	(0)	0	(0)	0
>50y	337	(85)	246	(169)	57	(21)	30	(7)	13	(3)	3	(1)	0

Figure S3

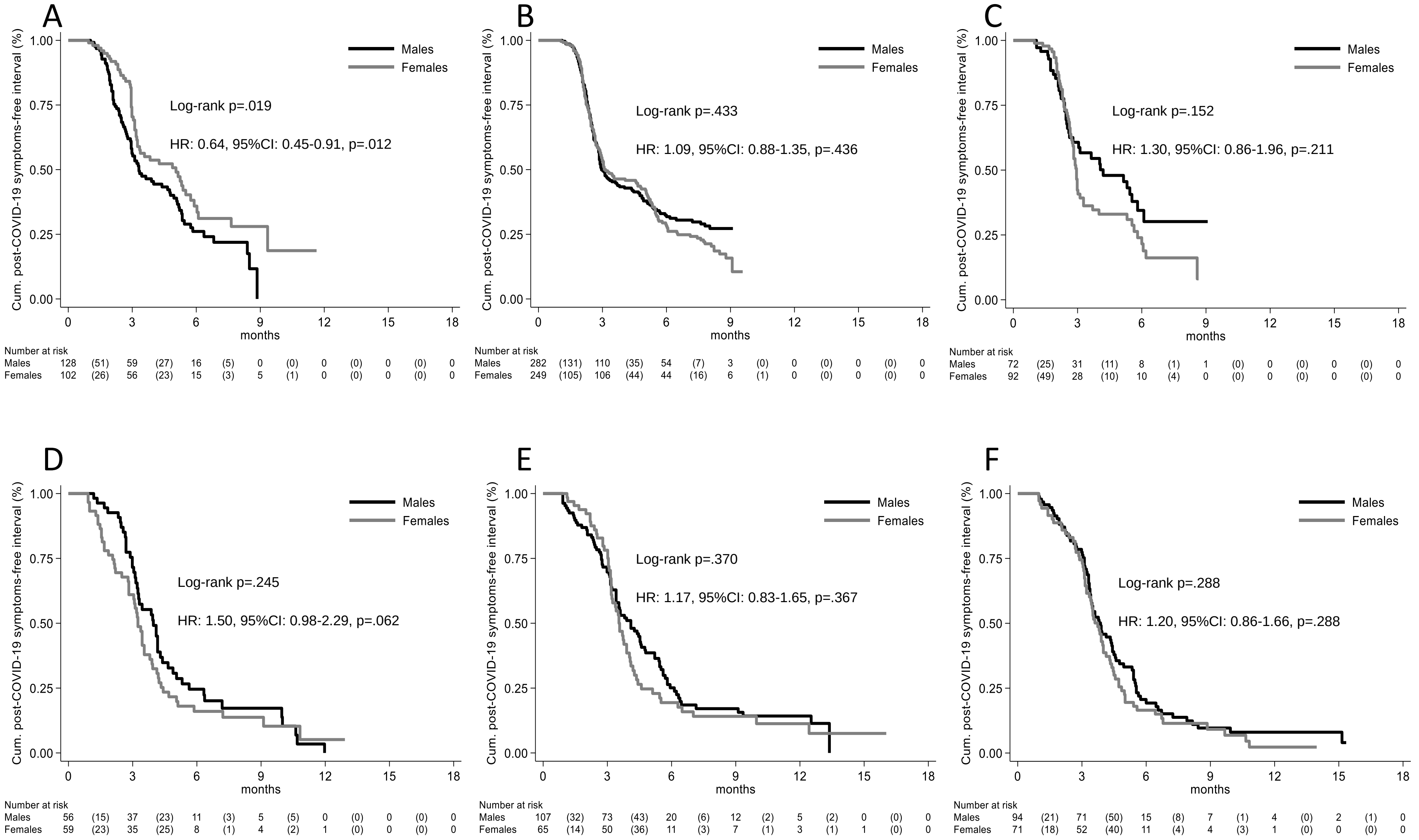
A
Males
Females
Log-rank p=.019
HR: 0.64, 95%CI: 0.45-0.91, p=.012
Cum. post-COVID-19 symptoms-free interval (%)
months
Number at risk
Males 128 (51) 59 (27) 16 (5) 0 (0) 0 (0) 0 (0) 0
Females 102 (26) 56 (23) 15 (3) 5 (1) 0 (0) 0 (0) 0
B
Males
Females
Log-rank p=.433
HR: 1.09, 95%CI: 0.88-1.35, p=.436
Cum. post-COVID-19 symptoms-free interval (%)
months
Number at risk
Males 282 (131) 110 (35) 54 (7) 3 (0) 0 (0) 0 (0) 0
Females 249 (105) 106 (44) 44 (16) 6 (1) 0 (0) 0 (0) 0
C
Males
Females
Log-rank p=.152
HR: 1.30, 95%CI: 0.86-1.96, p=.211
Cum. post-COVID-19 symptoms-free interval (%)
months
Number at risk
Males 72 (25) 31 (11) 8 (1) 1 (0) 0 (0) 0 (0) 0
Females 92 (49) 28 (10) 10 (4) 0 (0) 0 (0) 0 (0) 0
D
Males
Females
Log-rank p=.245
HR: 1.50, 95%CI: 0.98-2.29, p=.062
Cum. post-COVID-19 symptoms-free interval (%)
months
Number at risk
Males 56 (15) 37 (23) 11 (3) 5 (5) 0 (0) 0 (0) 0
Females 59 (23) 35 (25) 8 (1) 4 (2) 1 (0) 0 (0) 0
E
Males
Females
Log-rank p=.370
HR: 1.17, 95%CI: 0.83-1.65, p=.367
Cum. post-COVID-19 symptoms-free interval (%)
months
Number at risk
Males 107 (32) 73 (43) 20 (6) 12 (2) 5 (2) 0 (0) 0
Females 65 (14) 50 (36) 11 (3) 7 (1) 3 (1) 1 (0) 0
F
Males
Females
Log-rank p=.288
HR: 1.20, 95%CI: 0.86-1.66, p=.288
Cum. post-COVID-19 symptoms-free interval (%)
months
Number at risk
Males 94 (21) 71 (50) 15 (8) 7 (1) 4 (0) 2 (1) 0
Females 71 (18) 52 (40) 11 (4) 4 (3) 1 (0) 0 (0) 0

Figure S4

Category of symptom	Age class (years)	N	Hazard Ratio	95%CI	
Musculoskeletal	0 to 5	230	0.73	0.12	4.42
	6 to 11	531	0.79	0.47	1.33
	12 to 17	164	1.15	0.47	2.83
Cardiovascular	0 to 5	230	0.25	0.01	5.45
	6 to 11	531	0.97	0.40	2.41
	12 to 17	164	5.49	1.25	24.00
Respiratory	0 to 5	230	0.72	0.47	1.09
	6 to 11	531	0.89	0.67	1.19
	12 to 17	164	1.48	0.80	2.76
Neurological	0 to 5	230	1.18	0.51	2.75
	6 to 11	531	1.26	0.93	1.71
	12 to 17	164	1.88	1.08	3.27
Dermatological	0 to 5	230	0.50	0.19	1.29
	6 to 11	531	1.00	0.51	1.94
	12 to 17	164	0.94	0.22	4.04
Gastrointestinal	0 to 5	230	0.88	0.48	1.62
	6 to 11	531	0.91	0.62	1.33
	12 to 17	164	1.46	0.65	3.29
Sensory	0 to 5	230			
	6 to 11	531	1.44	0.32	6.45
	12 to 17	164	0.89	0.24	3.26
Sleep	0 to 5	230	0.49	0.23	1.02
	6 to 11	531	0.94	0.60	1.45
	12 to 17	164	1.79	0.93	3.47
Fatigue	0 to 5	230	0.44	0.14	1.40
	6 to 11	531	0.99	0.69	1.42
	12 to 17	164	1.64	0.90	2.97
Loss of appetite/weight loss	0 to 5	230	0.63	0.31	1.27
	6 to 11	531	1.25	0.79	1.99
	12 to 17	164	1.63	0.69	3.85
Any symptom	0 to 5	230	0.64	0.45	0.91
	6 to 11	531	1.09	0.88	1.35
	12 to 17	164	1.30	0.86	1.96

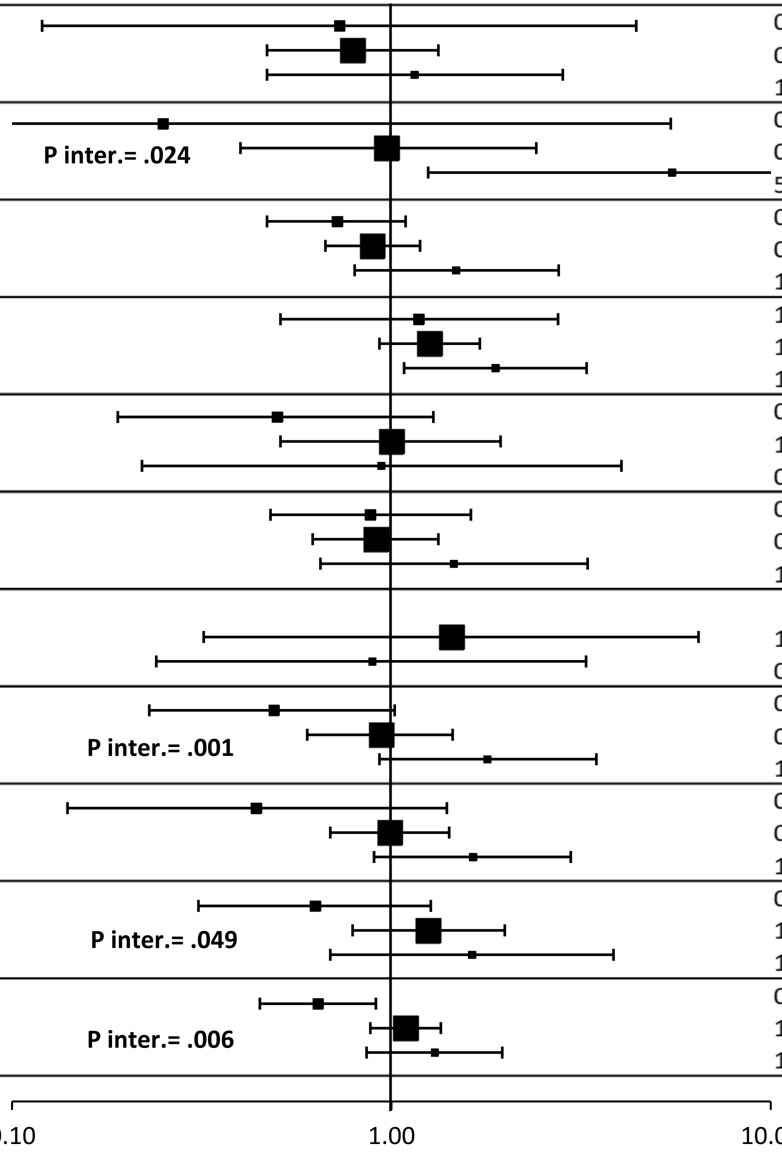

Figure S5

Category of symptom	Age class (years)	N	Hazard Ratio	95%CI	
Musculoskeletal	18 to 50	115	1.36	0.82	2.15
	51 to 64	172	1.00	0.67	1.48
	65+	165	1.46	0.99	2.16
Cardiovascular	18 to 50	115	2.54	1.40	4.64
	51 to 64	172	1.65	0.99	2.75
	65+	165	1.84	0.98	3.46
Respiratory	18 to 50	115	1.00	0.61	1.64
	51 to 64	172	1.02	0.68	1.52
	65+	165	1.73	1.16	2.58
Neurological	18 to 50	115	1.55	0.97	2.45
	51 to 64	172	1.23	0.84	1.79
	65+	165	1.41	0.96	2.05
Dermatological	18 to 50	115	1.20	0.54	2.64
	51 to 64	172	1.96	1.08	3.55
	65+	165	2.02	0.95	4.29
Gastrointestinal	18 to 50	115	2.39	1.33	4.32
	51 to 64	172	1.79	1.10	2.94
	65+	165	1.20	0.74	1.96
Sensory	18 to 50	115	1.91	0.70	5.21
	51 to 64	172	0.73	0.29	1.81
	65+	165	1.30	0.64	2.69
Sleep	18 to 50	115	1.86	1.01	3.45
	51 to 64	172	1.67	1.07	2.60
	65+	165	1.13	0.72	1.79
Fatigue	18 to 50	115	1.19	0.74	1.91
	51 to 64	172	1.07	0.73	1.59
	65+	165	1.43	0.98	2.09
Loss of appetite/weight loss	18 to 50	115	0.82	0.34	1.97
	51 to 64	172	1.84	0.92	3.69
	65+	165	1.57	0.87	2.82
Any symptom	18 to 50	115	1.50	0.98	2.29
	51 to 64	172	1.17	0.83	1.65
	65+	165	1.20	0.86	1.66

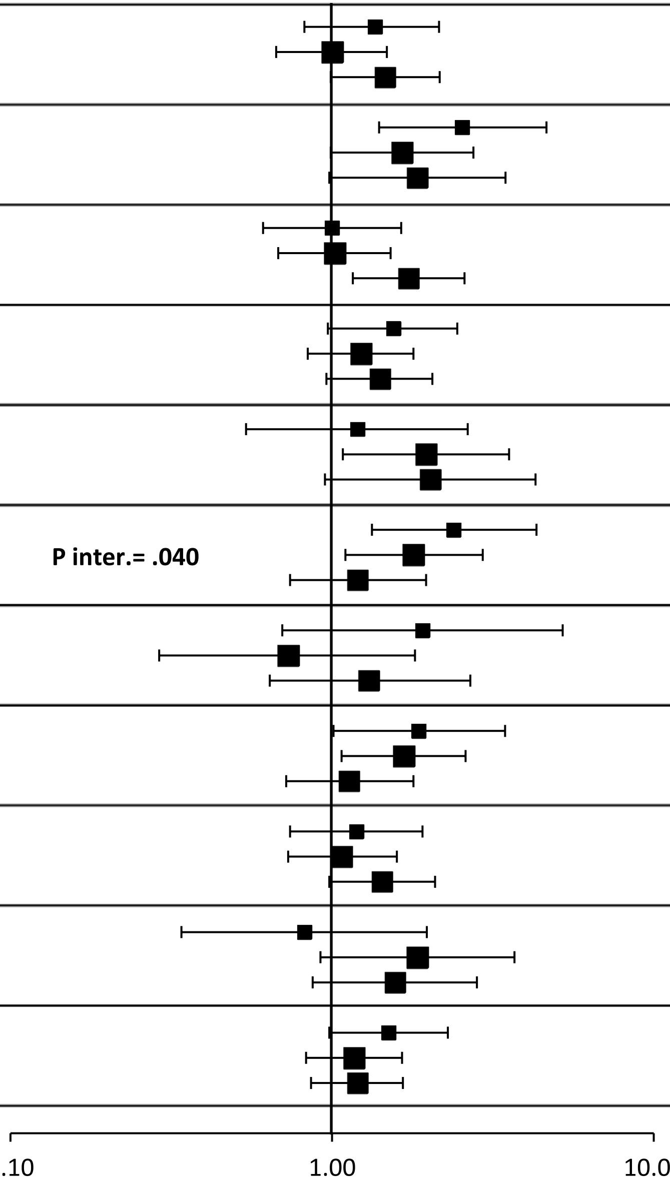

Figure S6

Category of symptom	Age class (years)	N	Hazard Ratio	95%CI	
Musculoskeletal	0-5	230	1.03	0.06	16.80
	6-11	531	0.52	0.25	1.10
	12-50	279	1.36	0.88	2.10
	>50	337	1.21	0.92	1.59
Cardiovascular	0-5	230	0.21	0.01	48.60
	6-11	531	1.49	0.42	5.33
	12-50	279	3.18	1.77	5.71
	>50	337	1.71	1.15	2.53
Respiratory	0-5	230	0.55	0.30	1.01
	6-11	531	1.12	0.74	1.70
	12-50	279	1.08	0.70	1.66
	>50	337	1.33	1.01	1.76
Neurological	0-5	230	1.75	0.56	5.44
	6-11	531	1.65	1.08	2.52
	12-50	279	1.63	1.11	2.39
	>50	337	1.30	1.00	1.69
Dermatological	0-5	230	0.32	0.06	1.61
	6-11	531	0.88	0.36	2.17
	12-50	279	1.28	0.61	2.71
	>50	337	1.95	1.23	3.11
Gastrointestinal	0-5	230	0.83	0.37	1.86
	6-11	531	1.18	0.71	1.98
	12-50	279	2.12	1.27	3.54
	>50	337	1.45	1.03	2.04
Sensory	0-5	230			
	6-11	531	1.01	0.06	16.30
	12-50	279	1.67	0.73	3.82
	>50	337	1.03	0.59	1.79
Sleep	0-5	230	0.49	0.17	1.41
	6-11	531	1.33	0.71	2.46
	12-50	279	2.33	1.39	3.93
	>50	337	1.41	1.02	1.93
Fatigue	0-5	230	0.47	0.04	5.26
	6-11	531	1.13	0.69	1.84
	12-50	279	1.36	0.91	2.03
	>50	337	1.24	0.99	1.62
Loss of appetite/weight loss	0-5	230	0.51	0.16	1.65
	6-11	531	0.81	0.41	1.57
	12-50	279	0.97	0.49	1.89
	>50	337	1.64	1.05	2.57
Any symptom	0-5	230	0.64	0.38	1.07
	6-11	531	1.40	1.04	1.88
	12-50	279	1.65	1.17	2.33
	>50	337	1.19	0.94	1.50

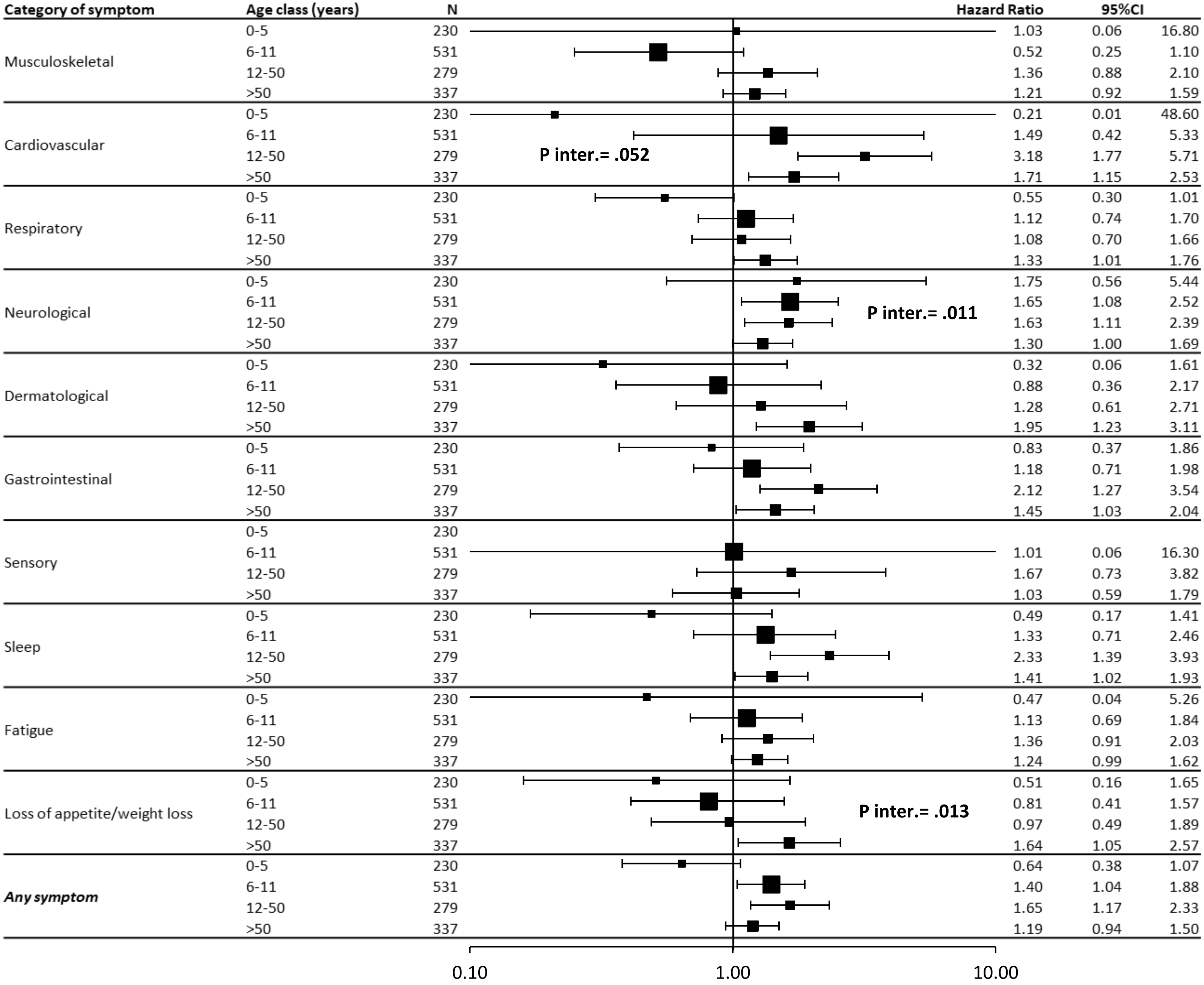

Figure S7

Category of symptom	Age class (years)	N	Hazard Ratio	95%CI	
Musculoskeletal	0 to 5	230	1.03	0.06	16.80
	6 to 11	531	0.52	0.25	1.10
	12 to 17	164	0.86	0.24	3.09
Cardiovascular	0 to 5	230	0.21	0.01	48.60
	6 to 11	531	1.49	0.42	5.33
	12 to 17	164	all 4 events in the female group		
Respiratory	0 to 5	230	0.55	0.30	1.01
	6 to 11	531	1.12	0.74	1.70
	12 to 17	164	1.58	0.56	4.46
Neurological	0 to 5	230	1.75	0.56	5.44
	6 to 11	531	1.65	1.08	2.52
	12 to 17	164	1.93	0.93	4.01
Dermatological	0 to 5	230	0.32	0.06	1.61
	6 to 11	531	0.88	0.36	2.17
	12 to 17	164	1.45	0.12	17.70
Gastrointestinal	0 to 5	230	0.83	0.37	1.86
	6 to 11	531	1.18	0.71	1.98
	12 to 17	164	1.08	0.38	3.08
Sensory	0 to 5	230	no events		
	6 to 11	531	1.01	0.06	16.30
	12 to 17	164	0.92	0.19	4.36
Sleep	0 to 5	230	0.49	0.17	1.41
	6 to 11	531	1.33	0.71	2.46
	12 to 17	164	3.41	1.13	10.30
Fatigue	0 to 5	230	0.47	0.04	5.26
	6 to 11	531	1.13	0.69	1.84
	12 to 17	164	1.31	0.58	2.95
Loss of appetite/weight loss	0 to 5	230	0.51	0.16	1.65
	6 to 11	531	0.81	0.41	1.57
	12 to 17	164	1.39	0.45	4.28
Any symptom	0 to 5	230	0.64	0.38	1.07
	6 to 11	531	1.40	1.04	1.88
	12 to 17	164	1.33	0.73	2.43

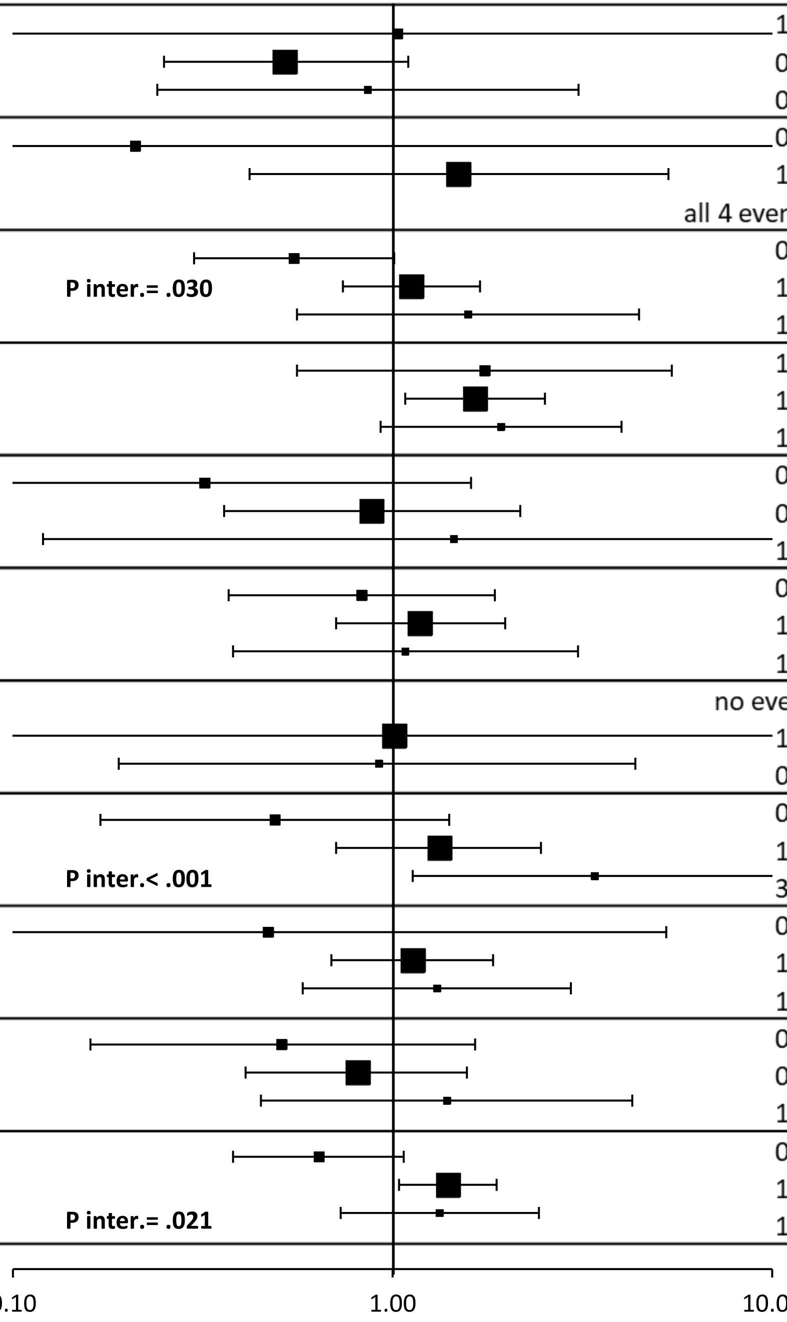

Supplement: Supplementary file 1 [file jcm-12-02924-s001.zip › jcm-2305590-supplementary.pdf]
